# Effect of COVID-19 Stimulus Payments in Mitigating Food Hardship

**DOI:** 10.1016/j.focus.2026.100502

**Published:** 2026-04-22

**Authors:** Oluwatosin O. Leshi, Meredith S. Duncan, Courtney Ortz, Suraksha Khanal, Brittany L. Smalls

**Affiliations:** 1 Department of Family Medicine and Community Health, University of Texas Medical Branch, Galveston, Texas; 2 Department of Biostatistics, University of Kentucky, Lexington, Kentucky

**Keywords:** COVID-19, food, food insecurity, SES, social determinants of health, social policy

## Abstract

**Introduction:**

The COVID-19 pandemic disrupted global economic and social systems, affecting food security, particularly among vulnerable individuals and households. Economic Impact Payments were introduced to provide financial relief and address the income gaps caused by pandemic-related economic disruptions.

**Methods:**

This study utilized Census Household Pulse Survey data from September 2020 to March 2021 to assess the effectiveness of Economic Impact Payments in mitigating food hardship among low-income households. Data from 494,433 participants meeting the eligibility criteria for Economic Impact Payments were obtained across 3 rounds of payments. Ordered logistic regression analysis was used to test the impact of Economic Impact Payments on food hardship during the COVID-19 pandemic.

**Results:**

Findings showed that the reduction in food hardship during the COVID-19 pandemic was associated with the Economic Impact Payment disbursement period, even after adjusting for age, sex, race/ethnicity, education level, marital status, employment status, income, and household composition. Key reasons for food hardship included an inability to afford additional food purchases, fear of going out, and the unavailability of desired food items.

**Conclusions:**

Economic Impact Payment disbursements were associated with reductions in food hardship during the COVID-19 pandemic, with similar benefits observed across racial, income, and employment groups. Developing strategic policies and mechanisms for timely and effective financial aid distribution is essential to mitigating similar challenges in the future.

## Highlights

• Economic Impact Payments were associated with reductions in food hardship during the COVID-19 pandemic.
• Broad, rapid cash transfers during crises mitigate food hardship in the U.S.
• Income-based eligibility yielded similar gains across groups, supporting equity.

## INTRODUCTION

Food insecurity, a critical determinant of health, exists when there is a limited or uncertain ability to acquire acceptable foods in socially acceptable ways.^1^ Food insecurity remains a concern in the U.S., with affected households increasing from 10.5% in 2020 to 13.5% in 2023, representing an estimated 47.4 million Americans struggling to access adequate food.^1^ In parallel, there has been renewed attention to measures of food sufficiency, which assess whether households generally have enough food to eat over a recent period and whether it is the kind of food they want. According to the U.S. Department of Agriculture (USDA), food sufficiency generally ranges from households reporting that they have enough of the kind of foods they want to eat (full food sufficiency) to those that sometimes or often do not have enough to eat, indicating food insufficiency and more severe food-related hardship.^1^ Households that report having enough to eat but not always the kinds of food they want are classified as having marginal food sufficiency, reflecting food-related problems and anxiety even when outright insufficiency is not present. Marginal food sufficiency and food insufficiency are food hardship experiences that represent forms of food insecurity and are known to increase the risk of chronic diseases and adverse mental health outcomes.^2^, ^3^, ^4^, ^5^, ^6^

The coronavirus disease 2019 (COVID-19) pandemic’s disruption of global economic and social systems has had lasting effects on food security, including food sufficiency. In the U.S., low-income households were disproportionately affected because the crisis deepened existing inequalities and amplified challenges in accessing adequate and nutritious food.^6^^,^^7^ The rates of food insecurity rose sharply since the onset of the pandemic in 2020, exacerbating disparities in food access among economically vulnerable populations and further worsening their vulnerability.^8^ During the early months of the pandemic, food insufficiency, reached levels as high as 19% in some surveys, driven by unemployment, disruptions in food supply chains, and rising food prices.^6^^,^^9^ Low-income households, particularly those headed by Black and Hispanic individuals and those with children, were disproportionately affected by these shocks.^7^^,^^10^

The U.S. government initiated Economic Impact Payments (EIPs) (stimulus checks) in April 2020 to provide financial relief to individuals and households facing economic hardship due to widespread disruption caused by COVID-19. These cash transfers were one of several social and economic policy interventions designed to help individuals and families bridge income gaps caused by pandemic-related economic disruptions and were received by over 80 million Americans.^11^^,^^12^ The stimulus payment amounts were calculated on the basis of household composition, with an average family of 4 receiving $3,400 total—specifically $1,200 per adult and $500 per qualifying child aged <17 years.^13^ The distribution method for stimulus checks varied depending on households’ tax filing status and banking arrangements. Understanding for whom these payments were made and whether they were associated with improvement in food-related harship hunger policies during health and economic crises.

In this study, national survey data were analyzed to assess whether stimulus check payments were associated with reductions in food hardship among low-income households across different demographic groups during the COVID-19 pandemic. Food hardship was operationalized as any report of less than full food sufficiency on the USDA food sufficiency item. This analytic construct of food hardship is therefore broader than the USDA’s standard food insecurity categories and should be interpreted accordingly. This approach allows to capture a spectrum of food-related challenges ranging from marginal food sufficiency to outright food insufficiency relevant to the economically vulnerable populations most affected by the pandemic. Understanding these dynamics is crucial for informing future policy decisions aimed at enhancing food security and addressing the needs of economically vulnerable populations during health and economic crises.

## METHODS

### Study Sample

Data from Weeks 16–27 (September 2020–March 2021) of the Household Pulse Survey (HPS) were used to assess the association between the disbursement of EIPs and food hardship. The HPS is a 20-minute cross-sectional online survey collaboratively developed by the USDA Economic Research Service, Bureau of Labor Statistics, National Center for Health Statistics, National Center for Education Statistics, and the Department of Housing and Urban Development. The survey was conducted by the U.S. Census Bureau to assess how emerging social and economic issues affect households across the U.S. Participants in the HPS are non-institutionalized adults (aged ≥18 years) from randomly selected households across the U.S. In conducting the HPS, the Census Bureau’s Master Address File was used to sample households. Selected households were contacted through email or text, and responses were collected through the Qualtrics online platform. Sample weights were applied to adjust for nonresponse bias and to ensure representativeness. Detailed, deidentified study data are publicly available on the Census Bureau's website.

From the base sample of 916,651 HPS respondents during Weeks 16–27, the sample was narrowed to 498,998 respondents who were eligible to receive EIP on the basis of their marital status and 2019 household income: unmarried individuals were eligible for EIP if their 2019 pretax income was less than $75,000, whereas married individuals were eligible for EIP if their 2019 pretax household income was less than $150,000. From this sample, individuals with missing data on the outcome (*n*=1,083) and on covariates (*n*=3,482) were sequentially excluded, yielding a final analytic sample of 494,433. This study was exempt from IRB regulations because the data were deidentified and publicly available.

### Measures

In this analysis, the completion of the HPS during a period in which EIPs were (or were not) disbursed served as the exposure of interest. Specifically, responses provided in Weeks 16–21 of the HPS (September 30, 2020–December 21, 2020) were considered unexposed to EIP receipt, whereas responses provided in the next 6 weeks (Weeks 22–27 of the HPS; January 6, 2021–March 29, 2021) were considered exposed to EIP receipt. The second round of EIP was issued between December 29, 2020 and January 15, 2021, whereas the third round was disbursed beginning on March 12, 2021.

Food hardship was used as the outcome of interest because it captures the full spectrum of households affected by COVID-19–related disruptions. This includes those experiencing marginal food security as well as outright food insufficiency, thereby providing a more sensitive measure of how EIP receipt may have mitigated food-related hardship during the pandemic. Food hardship was defined using the USDA 7-day food insufficiency item; households reporting *sometimes* or *often not enough to eat* were considered to have food insufficiency, reflecting severe food hardship. In contrast, those reporting *enough, but not always the kinds of foods wanted,* were included to capture milder forms of food-related hardship.

Participants were asked, *In the last 7 days, which of these statements best describes the food eaten in your household?* with 4 potential responses: (1) *enough of the kinds of food (I/we) wanted to eat*; (2) *enough, but not always the kinds of food (I/we) wanted to eat*; (3) *sometimes not enough to eat*; and (4) *often not enough to eat*. In the primary models, this 4-level ordinal variable served as the outcome of interest. In secondary analyses, a binary version of this variable was created, where individuals who responded *enough of the kinds of food (I/we) wanted to eat* were considered to have enough food, whereas those who responded with any of the other 3 responses were considered to have some form of food insecurity.

Covariates were self-reported and included age, sex, race/Hispanic ethnicity, education, 2019 pretax household income, marital status, number of children in the household, number of adults in the household, past-week employment, participation in Supplemental Nutrition Assistance Program (SNAP) or food stamp benefits, total amount spent on food in the last week, and whether anything other than income (e.g., savings, selling assets, credit cards, loans, borrowing from others, and others) was used to pay for necessities in the last week. The specific coding for these variables is detailed in Appendix Table 1 (available online).

Participants missing data on the outcome or covariates (except for total amount spent on food in the last week and use of non-income sources to pay for past-week necessities) were excluded, as mentioned earlier; these variables had <1% missingness. In contrast, data on the total amount spent on food in the last week and/or the use of non-income sources to pay for past-week necessities were missing for an additional 32,100 individuals.

To handle missingness in these variables in regression analyses, missing-data indicators were added (for both variables), and missing values were set equal to the mean of the observed values (total amount spent on food in the last week), so that missing values would not exert leverage on the variable’s estimate of effect.

In regression analyses, missingness was addressed by including missing-data indicators for both variables and imputing missing values with the mean of the observed values (total weekly food expenditure), thereby minimizing their influence on effect estimates.

### Statistical Analysis

The means and SEs of continuous variables were obtained, along with frequencies and percentages of categorical variables stratified by period. Differences in summary statistics across periods were assessed through *t*-tests for continuous variables or Rao–Scott chi-square tests for categorical variables. Next, the frequencies of various reasons for food hardship, both overall and stratified by period, were calculated. Then, the weekly frequency of each level of food hardship was plotted. Ordinal (i.e., ordered) logistic regression was used to assess the association between food hardship and responding in periods with or without EIP receipt, and it was confirmed that the proportional odds assumption was not severely violated by testing for differential effects of the exposure across cumulative levels of food hardship. Four sets of models were fitted: (1) unadjusted; (2) adjusted for age, sex, race/ethnicity, education, marital status, and employment in the last 7 days; (3) adjusted for covariates in Model 2 plus household income and total number of adults and children in the household; and (4) adjusted for covariates in Model 3 plus SNAP participation, total amount spent on food in the last 7 days, and whether nonincome sources were used to pay for necessities in the last 7 days, along with the missing data indicators for the last 2 variables. Heterogeneity in the association between food hardship and responding in periods with or without EIP receipt was assessed by race/ethnicity, income, and employment in the last 7 days by including interaction terms between the period and each of these variables in Model 2. In sensitivity analyses, these 4 models were repeated using standard logistic regression, with a binary indicator of food hardship as the outcome.

All analyses were conducted in SAS 9.4 (Cary, NC) using procedures designed for the analysis of complex survey data. Individual rather than household weights along with 80 replicate weights were used. An indicator of whether individuals were in the analytic sample (i.e., met the income requirement for receipt of EIP and had no missing data) was created and included as a domain variable in the models so that the SEs were estimated correctly. A 2-sided *p*<0.05 was considered statistically significant for the assessment of main effects; interactions were considered statistically significant at a 2-sided *p*<0.1 to avoid overlooking potentially meaningful effect modifications.

## RESULTS

The analytic sample for this study included data from 494,433 individuals—248,248 who responded before the disbursement of Rounds 2 and 3 of EIP and 246,185 who responded during or after the disbursement of Rounds 2 and 3 of EIP. Respondents were aged 50 years on average, were slightly more likely to be female and married, were predominantly non-Hispanic White, and were more likely to have less than a college education (Table 1). Approximately half of the sample had a household income <$50,000; only 54% had been employed in the last 7 days; and approximately 58% of the sample had used non-income sources to cover necessary expenses in the last 7 days, but only 14% of respondents participated in and received SNAP or food stamp benefits. The proportion of participants who relied on non-income sources (such as savings or borrowing) to meet essential needs was higher before EIP receipt but was significantly reduced during EIP disbursement (*p*=0.03).

**Table 1:** Sample Characteristics Stratified^a^ by Period Defined by EIP Disbursement (N=494,433)

Characteristic	Overall (N=494,433)	Period without EIP (*n*=248,248)	Period with EIP (*n*=246,185)	*p*-value
Age, years^a^	49.98 (0.18)	49.87 (0.26)	50.10 (0.25)	0.44
Sex				
Male	190,731 (46.44)	95,732 (46.38)	94,999 (46.50)	0.90
Female	303,702 (53.55)	152,516 (53.61)	151,186 (53.49)	
Race/ethnicity				0.95
Asian	17,829 (4.03)	8,6,46 (3.94)	9,183 (4.11)	
Black	34,488 (11.25)	17,016 (11.14)	17,472 (11.37)	
Hispanic	46,303 (17.03)	22,137 (16.92)	24,166 (17.13)	
Other or multiracial	18,963 (3.83)	9,853 (3.93)	9,110 (3.72)	
White	376,850 (63.85)	190,596 (64.04)	186,254 (63.65)	
Highest level of education				0.96
Less than high school	3,242 (2.61)	1,515 (2.42)	1,727 (2.79)	
Some high school	7,050 (5.59)	3,421 (5.41)	3,629 (5.79)	
High-school graduate or equivalent	64,912 (33.54)	32,460 (33.80)	32,452 (33.26)	
Some college	121,056 (21.78)	60,969 (21.92)	60,087 (21.65)	
Associate’s degree	61,117 (10.43)	30,659 (10.49)	30,458 (10.38)	
Bachelor’s degree	137,343 (15.64)	69,349 (15.49)	67,994 (15.78)	
Graduate degree	99,713 (10.39)	49,875 (10.45)	49,838 (10.32)	
Married	299,440 (59.73)	150,187 (60.04)	149,253 (59.43)	0.61
Household income, U.S. $				0.93
<25,000	69,033 (18.53)	34,778 (18.26)	34,255 (18.80)	
25,000–34,999	59,346 (14.47)	29,733 (14.44)	29,613 (14.49)	
35,000–49,999	76,288 (16.56)	37,997 (16.41)	38,291 (16.71)	
50,000–74,999	123,460 (23.44)	61,410 (23.34)	62,050 (23.55)	
75,000–99,999	68,211 (11.95)	34,556 (12.31)	33,655 (11.59)	
≥100,000	98,905 (15.04)	49,774 (15.23)	48,321 (14.85)	
Employment in last 7 days	268,560 (53.49)	138,897 (53.62)	129,663 (53.37)	0.85
Number of adults in household^a^	2.56 (0.06)	2.55 (0.09)	2.58 (0.07)	0.55
Number of children in household^a^	0.70 (0.02)	0.70 (0.02)	0.70 (0.02)	0.77
Amount spent on food/week, U.S. $^a^	276 .42 (3.86)	272.61 (4.95)	280.30 (4.41)	0.16
SNAP or food stamp receipt	45,715 (13.81)	23,565 (13.73)	22,150 (13.91)	0.82
Nonincome source(s) used to cover Necessary expenses	262,632 (48.44)	136,617 (60.00)	126,015 (56.84)	0.03

a Continuous variable; summary statistics presented as survey-weighted mean and SE.

Participants who reported any form of food hardship were asked to indicate their reasons for this condition. Regardless of the period, half of the respondents reported that they could not afford to buy more food, whereas another 33% said that they were afraid or did not want to go out to buy more food (Table 2). During the period before EIP disbursement, 36% of people reported that stores did not have the food that they wanted; this proportion dropped to 27% during the period when EIPs were disbursed. Less common reasons for food hardship were that people were not able to get out to buy food (14%) or were not able to get groceries or meals delivered (6%).

**Table 2:** Reasons for Food Hardship

Reason for food hardship^a^	Overall (N=178,126)	Period without EIP (*n*=96,187)	Period with EIP (*n*=81,939)
Couldn’t afford to buy more food	88,135 (49.47)	48,155 (50.06)	39,980 (48.79)
Afraid to go or didn’t want to go out to buy food	58,187 (32.68)	30,735 (31.95)	27,452 (33.50)
The stores did not have the foods I wanted	56,752 (31.86)	34,353 (35.71)	22,399 (27.39)
Couldn’t get out to buy food	25,499 (14.32)	13,186 (13.71)	12,313 (15.01)
Couldn’t get groceries or meals delivered to me	11,303 (6.34)	6,001 (6.24)	5,302 (6.46)
None of the above	9,958 (5.59)	5,036 (5.24)	4,922 (6.01)

*Note*: Values are displayed as *n* (%).

EIP, Economic Impact Payment.

a Respondents chose as many reasons as applicable.

**Figure 1:**
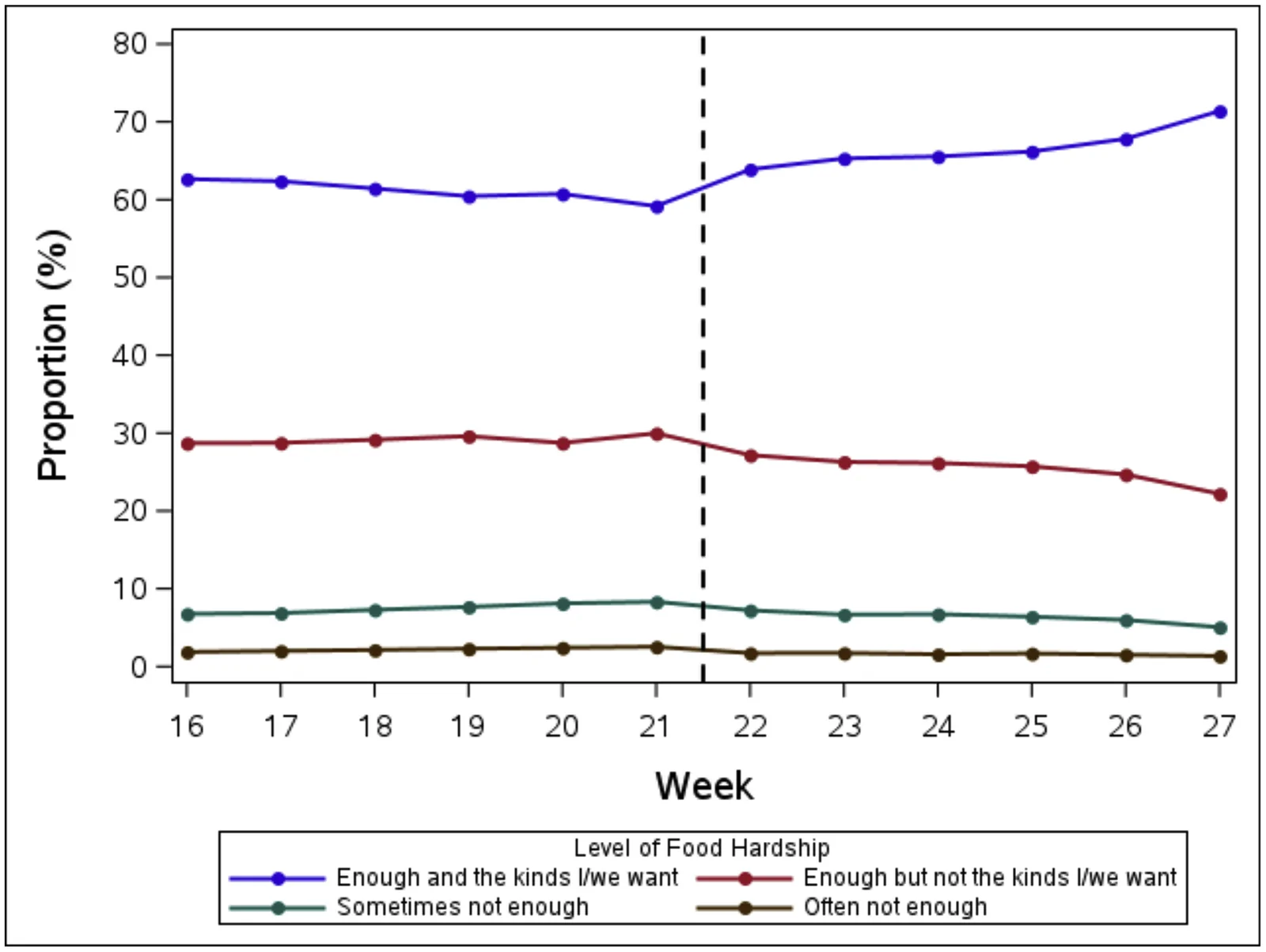
Plot of food hardship by week.

When plotting the frequency of each level of food hardship by week, there was a noticeable and marked uptick in the proportion of individuals reporting having enough food in Weeks 22–27—the period during which Rounds 2 and 3 of the EIP were disbursed—compared with the proportion in Weeks 16–21 (Figure 1). The study also reported a 15%–18% reduction in the odds of experiencing food hardship during the period of EIP disbursement compared with the odds in the non-EIP period (Table 3).

**Table 3:** Association Between Period of EIP Disbursement and Food Hardship

Model	OR (95% CI)	*p*-value
Unadjusted	0.85 (0.76, 0.95)	0.0051
Multivariable adjusted 1^a^	0.83 (0.74, 0.93)	0.0022
Multivariable adjusted 2^b^	0.82 (0.73, 0.92)	0.001
Multivariable adjusted 3^c^	0.85 (0.76, 0.94)	0.0032

a Includes adjustment for age, sex, race/ethnicity, education status, marital status, and employment in last 7 days.

b Includes adjustment for all covariates listed in (a) plus household income and total number of adults in household and children in household.

c Includes adjustment for all covariates listed in (b) plus participation in SNAP or food stamp benefits, total amount spent on food in last week, whether anything other than income used to pay for necessities, and missing data indicators for the final 2 variables. EIP, Economic Impact Payment; SNAP, Supplemental Nutrition Assistance Program.

This association was relatively stable, regardless of which set of covariates was included in the model. No significant interaction was observed between the period of response and race/ethnicity (*p*=0.95), household income (*p*=0.99), or employment in the last 7 days (*p*=0.83). Still, across the full period of analysis, it was noted that non-White individuals, those with lower incomes, and individuals who were unemployed in the last 7 days experienced greater food hardship than White individuals, those with higher incomes, and employed individuals, respectively (Appendix Figure 1, available online). In sensitivity analyses that dichotomized food hardship, the results were consistent, with those who replied during the EIP disbursement period having significantly lower food hardship rates than those who replied before the EIP disbursement (Appendix Table 2, available online).

## DISCUSSION

This study provides important insights into the dynamics of food hardship during the COVID-19 pandemic. Half of the participants reported food hardship due to an inability to afford more food, whereas another 33% cited fear or reluctance to go out to buy food. This aligns with prior research suggesting that economic hardship and public health concerns jointly exacerbate food insufficiency.^14^ Therefore, it underscores that food hardship is not solely a matter of financial constraints but is also influenced by other factors, such as lack of market access and fear of public spaces during the pandemic. Before the disbursement of EIPs, 36% of participants reported that stores did not have the food that they wanted, a proportion that dropped to 27% during the EIP disbursement period. This reduction reflects not only the alleviation of financial barriers faced by economically vulnerable individuals through direct cash assistance but also improvements in broader supply chain disruptions that affected food distribution and availability during the pandemic. Pandemic-related shutdowns, travel restrictions, labor shortages, and transportation challenges led to initial food shortages and stockouts; as the food industry adapted and supply chains stabilized, both food availability and varieties improved, thereby increasing options for food choice.^15^^,^^16^ Findings from this study support previous findings showing the effect of financial resources, such as unemployment insurance, on food access and food availability.^17^ Although EIPs likely helped improve food access by increasing household purchasing power, the simultaneous decline in reported shortages also mirrors concurrent contextual improvements in store inventories and reduced pandemic-related disruptions.

Although EIP disbursements alleviated financial barriers, they were not enough to address food hardship, because households with food hardship continued to rely on other sources of food assistance. A study found that recipients of the first round of EIP continued to depend on additional resources, such as food pantries, and experienced a 9.2 percentage point reduction in their likelihood of food insufficiency.^18^ This study aligns with an earlier study that indicated that EIPs helped households reduce reliance on nonincome sources.^19^ In this study, the proportion of individuals using non-income sources to cover necessary expenses decreased significantly during EIP receipt. This suggests that before receiving EIPs, many individuals and households that experienced income loss during COVID-19 relied on nonincome sources. The lump-sum EIP disbursement likely increased their available resources and substituted for many of the nonincome sources of support.

In this study, the proportion of individuals who reported having enough food increased after EIP disbursement, and those who responded during the EIP period had a 15%–18% reduction in the odds of experiencing food hardship compared with participants before receiving the stimulus checks. This finding demonstrates that EIPs could be linked to improved food sufficiency during the disbursement period, suggesting that timely financial support helps individuals and households meet their food needs and overcome any anxieties due to a lack of access. However, evidence from recent studies shows that although 1-time payments can create an immediate reduction in food hardship, their effects tend to diminish over time as the funds are used up.^20^^,^^21^ The reduction observed in this study supports the idea that ongoing financial assistance may be more effective at consistently alleviating immediate food hardships during economic downturns than 1-time disbursements. Furthermore, the observed reductions in food hardship during EIP disbursements indicate that recipients likely prioritized their EIP funds for essential needs, such as food. This observation is consistent with previous research demonstrating that people primarily allocate their stimulus payments to food rather than to nonessential needs.^12^^,^^22^^,^^23^ This highlights the adaptive spending behaviors of economically vulnerable populations and underscores the value of direct cash transfers in supporting basic needs and addressing food hardship.

Although the prevalence of lost income during the pandemic was similar across most racial and ethnic groups, population groups such as Black/African American, Indigenous, Spanish-speaking Latino, and Native Hawaiian/Pacific Islander households were less equipped to absorb economic shocks.^24^ In this study, non-White participants, those with lower incomes, and those who were unemployed experienced higher levels of food hardship. This finding is consistent with prior work showing that race, income, and employment status shape vulnerability to food insufficiency and hardship.^24^, ^25^, ^26^ However, the study found no evidence that the short-term association between EIP receipt and reductions in food hardship differed by race, income, or employment status, indicating that income-based EIP distribution was similarly effective across these groups in the period studied. Other studies indicate that many households continued to rely on additional food assistance, underscoring that EIPs alone cannot fully address food hardship.^18^^,^^27^ Taken together, these findings suggest that broad cash transfers can reduce food hardship across diverse groups but must be accompanied by other policies that address structural inequities in wealth and access to resources. The findings that broad, income-based EIP distribution was associated with reduced food hardship, with no evidence of differential effects by race, income, or employment status, align with the observation in an earlier study^28^ that stimulus payments increased overall spending among low-income populations. Although transaction data were used by Li and colleagues^28^ to highlight variation in where stimulus funds were spent and discuss the potential value of geo- and category-targeted designs, the null interaction results suggest that, at least for food hardship, broad income-based eligibility produced comparable benefits across demographic and socioeconomic groups.

On the basis of the findings from this study, targeted approaches based on income-based distribution should be considered in future economic relief policies. This study joins other studies in advocating for broader social intervention programs to achieve better outcomes through higher take-up, reduced stigma, and stronger political sustainability.^28^, ^29^, ^30^

### Limitations

This study has several limitations. First, the HPS has a consistently low response rate and relies on online data collection, raising concerns about nonresponse and coverage bias and whether the sample fully represents the U.S. population. Although Census weighting procedures are designed to improve representativeness, households without reliable internet access, a cell phone number, or an email address are less likely to be included, potentially underestimating food hardship among the most marginalized groups.

Second, the primary outcome, food hardship, was constructed from the USDA 7-day food insufficiency item and does not correspond exactly to the USDA’s standard multi-item food security categories. Food hardship was defined as reporting less than full food sufficiency (responses 2–4 on the insufficiency item), with responses of *sometimes* or *often not enough to eat* indicating food insufficiency (more severe hardship) and *enough, but not always the kinds of food we wanted to eat* capturing milder constraints on dietary quality and choice. This broader construct allows the inclusion of households facing both marginal food security and outright insufficiency but may limit direct comparability with studies that use the full USDA food security modules.

Third, all measures were self-reported and may be subject to social desirability and recall bias, which could affect the accuracy of reported food hardship, income loss, and the use of nonincome sources. Beyond EIPs, other economic and social interventions, such as the temporary expansion of the Child Tax Credit, the increase in SNAP benefits, local guaranteed income, and emergency assistance programs, were also implemented during the study period and likely contributed to the observed improvements in food hardship. As a result, the associations observed between EIP timing and food hardship should be interpreted in the context of these overlapping policies rather than as an isolated effect of EIPs.

Finally, this study used mean imputation along with missing data indicators to account for missing values, which may have biased the results if the data were missing not at random. However, in this case, it seems reasonable to assume that the data are, at most, missing at random, because the analysis accounted for both income and receipt of SNAP benefits in the models—both of which are likely correlated with the missing values of total amount spent on food and use of nonincome sources to pay for necessities in the prior week. Thus, this bias is likely to be small.

## CONCLUSIONS

In conclusion, the receipt of EIPs was associated with meaningful reductions in food hardship during the COVID-19 pandemic, with no evidence of differential effects by race, income, or employment status in this study. These findings support the use of broad, income-based cash transfer policies during large-scale economic disruptions to mitigate food hardship across diverse population groups. Consistent with prior work indicating that EIPs and other cash support programs reduce food hardship but do not fully eliminate it, the results from this study suggest that such broad payments are vital but not sufficient components of a comprehensive safety net. Future research should examine how payment timing, frequency, and coordination with existing safety net programs can enhance and sustain reductions in food hardship and assess whether any additional targeting beyond income-based eligibility improves impacts without undermining the equity and administrative simplicity that broad distribution provides.

## CRediT authorship contribution statement

**Oluwatosin O. Leshi:** Conceptualization, Writing – original draft, Writing – review & editing. **Meredith S. Duncan:** Formal analysis, Writing – original draft. **Courtney Ortz:** Writing – original draft. **Suraksha Khanal:** Formal analysis, Writing – original draft. **Brittany L. Smalls:** Conceptualization, Writing – review & editing.

## Appendix Supplementary materials

Image, application 1

## Footnotes

Supplementary material associated with this article can be found in the online version at doi:10.1016/j.focus.2026.100502.

## References

[1] Measurement of food security in the U.S. United States Department of Agriculture, Economic Research Service.https://www.ers.usda.gov/topics/food-nutrition-assistance/food-security-in-the-us/measurement#comparison. Updated March 30, 2026. Accessed January 25, 2025.

[2] Pooler J.A., Hartline-Grafton H., DeBor M., Sudore R.L., Seligman H.K. (2019). Food insecurity: a key social determinant of health for older adults. J Am Geriatr Soc.

[3] Nagata J.M., Palar K., Gooding H.C., Garber A.K., Bibbins-Domingo K., Weiser S.D. (2019). Food insecurity and chronic disease in U.S. young adults: findings from the National Longitudinal Study of Adolescent to Adult Health. J Gen Intern Med.

[4] Pan L., Sherry B., Njai R., Blanck H.M. (2012). Food insecurity is associated with obesity among U.S. adults in 12 states. J Acad Nutr Diet.

[5] Geng J., Yu X., Bao H. (2021). Chronic diseases as a predictor for severity and mortality of COVID-19: a systematic review with cumulative meta-analysis. Front Med (Lausanne).

[6] Nagata J.M., Ganson K.T., Whittle H.J. (2021). Food insufficiency and mental health in the U.S. during the COVID-19 pandemic. Am J Prev Med.

[7] Kim-Mozeleski J.E., Pike Moore S.N., Trapl E.S., Perzynski A.T., Tsoh J.Y., Gunzler D.D. (2023). Food insecurity trajectories in the U.S. during the first year of the COVID-19 pandemic. Prev Chronic Dis.

[8] Schanzenbach D., Pitts A. (Published 2020). https://www.ipr.northwestern.edu/documents/reports/ipr-rapid-research-reports-pulse-hh-data-10-june-2020.pdf.

[9] Li Y., Li D., King C. (2022). Food insufficiency among job-loss households during the pandemic: the role of food assistance programs. Sustainability.

[10] Altman C.E., Dondero M., Heflin C.M., Nusbaum D. (2022). Current and future food insufficiency during COVID-19: examining disparities by race/ethnicity and recent work loss. J Racial Ethn Health Disparities.

[11] Baker S.R., Farrokhnia R.A., Meyer S., Pagel M., Yannelis C. (2023). Income, liquidity, and the consumption response to the 2020 economic stimulus payments. Rev Financ.

[12] Coibion O, Gorodnichenko Y, Weber M. How did U.S. consumers use their stimulus payments? *SSRN J*. Preprint. Online August 17, 2020. 10.2139/ssrn.3675543.

[13] Jacobs M., McDade T.R., Chaparro M.V., Corea M. (2023). Sick, hungry, and vulnerable: federal stimulus and food security on marginalized populations during the COVID-19 pandemic. J Racial Ethn Health Disparities.

[14] Gómez-Corona C., Ramaroson Rakotosamimanana V.R., Sáenz-Navajas M.P. (2021). To fear the unknown: COVID-19 confinement, fear, and food choice. Food Qual Prefer.

[15] Xu Z., Elomri A., Kerbache L., El Omri A. (2020). Impacts of COVID-19 on global supply chains: facts and perspectives. IEEE Eng Manag Rev.

[16] Jasmine Chang A.J., Zhou F., El-Rayes N., Shi J. (2024). Food transportation and price impacted by diesel price and truck-driver shortage pre-, amid and post pandemic. Transp Res E.

[17] Raifman J., Bor J., Venkataramani A. (2021). Association between receipt of unemployment insurance and food insecurity among people who lost employment during the COVID-19 pandemic in the United States. JAMA Netw Open.

[18] Wahdat A.Z. (2022). Economic impact payments and household food insufficiency during COVID-19: the case of late recipients. Econ Disaster Clim Chang.

[19] Parker J.A., Schild J., Erhard L., Johnson D.S. (2022). Economic impact payments and household spending during the pandemic. Brookings Pap Econ Act.

[20] Altındağ O., O’Connell S.D. (2023). The short-lived effects of unconditional cash transfers to refugees. J Dev Econ.

[21] Parolin Z., Ananat E., Collyer S., Curran M., Wimer C. (2023). The effects of the monthly and lump-sum child tax credit payments on food and housing hardship. AEA Pap Proc.

[22] Asebedo S.D., Liu Y., Gray B., Quadria T.H. (2020). How Americans used their COVID-19 economic impact payments. Fin Plan Rev.

[23] Bina J.D., Tonsor G.T., Briggeman B.C. (2023). COVID-19 federal aid and household food expenditures. J Agric Appl Econ.

[24] Alhomsi A., Quintero S.M., Ponce S. (2023). Racial/ethnic disparities in financial hardship during the first year of the pandemic. Health Equity.

[25] Gundersen C., Ziliak J.P. (2015). Food insecurity and health outcomes. Health Aff (Millwood).

[26] Quintero Arias C., Rony M., Jensen E. (2024). Food insecurity in high-risk rural communities before and during the COVID-19 pandemic. Heliyon.

[27] Harper K., Belarmino E.H., Acciai F., Bertmann F., Ohri-Vachaspati P. (2022). Patterns of food assistance program participation, food insecurity, and pantry use among U.S. households with children during the COVID-19 pandemic. Nutrients.

[28] Li K., Foutz N.Z., Cai Y., Liang Y., Gao S. (2021). Impacts of COVID-19 lockdowns and stimulus payments on low-income population’s spending in the United States. PLoS One.

[29] Wilson B., Tate E., Emrich C.T. (2021). Flood recovery outcomes and disaster assistance barriers for vulnerable populations. Front Water.

[30] Remler D.K., Glied S.A. (2003). What other programs can teach us: increasing participation in health insurance programs. Am J Public Health.

