# Supplementary material for: Effect of COVID-19 Stimulus Payments in Mitigating Food Hardship
Source: AJPM Focus. 2026 Apr 22;5(5):100502. doi: 10.1016/j.focus.2026.100502 (PMC13519611; doi:10.1016/j.focus.2026.100502)
Supplement: Supplementary file 1 [file mmc1.docx]

**Title:** Effect of COVID-19 Stimulus Payments in Mitigating Food Hardship

**Authors:** Oluwatosin Leshi, PhD, MSc, Meredith Duncan, PhD, Courtney Ortz, PhD, Suraksha Khanal, MPH, RN, Brittany L. Smalls, PhD, MHSA, MSHPsych

**Journal:** AJPM Focus

**Appendix Table 1:** Covariate Definitions

| **Variable** | **Coding** |
| --- | --- |
| Age | Approximated as difference between year of response and self-reported birth year |
| Sex | Male/Female |
| Race/Ethnicity | Non-Hispanic White, Alone  Non-Hispanic Black, Alone  Non-Hispanic Asian, Alone  Hispanic  Any other race alone or in combination |
| Highest Education | Less than high school  Some high school  High School graduate or equivalent (e.g., GED)  Some college but no degree or in progress  Associate’s degree  Bachelor’s degree  Graduate degree |
| 2019 Pre-Tax Household Income | Less than $25,000  $25,000 - $34,999  $35,000 - $49,999  $50,000 - $74,999  $75,000 - $99,999  $100,000 - $149,999 |
| Marital Status | Married  Not Married |
| Number of Children in the Household | Total people under 18 in household (range 0-5) |
| Number of Adults in the Household | Total people 18 or older in household (range 1-10) |
| Past-week Employment | Any work in last 7 days for pay or profit (yes/no) |
| Participation and receipt of SNAP Benefits | Receipt of SNAP or Food Stamps for anyone in household (yes/no) |
| Total Amount Spent on Food in Last week | Sum of money spent in the last 7 days on food to be prepared and eaten at home and money spent in the last 7 days on prepared food (fast food, restaurants, carry out, delivery). |
| Non-income used to pay for necessities | Indicator of whether individuals used credit cards or loans, money from savings or selling assets, borrowing from family or friends, unemployment insurance benefit payments, money saved from deferred or forgiven payments, or SNAP to pay for necessities (yes/no) |

**Appendix Table 2:** Association between Period of EIP Disbursement and Food Hardship (Binary)

| **Model** | **Odds Ratio [95% CI]** | **p-value** |
| --- | --- | --- |
| Unadjusted | 0.84 [0.75, 0.94] | 0.0026 |
| Multivariable Adjusted 1^a^ | 0.82 [0.73, 0.92] | 0.0011 |
| Multivariable Adjusted 2^b^ | 0.81 [0.71, 0.91] | 0.0006 |
| Multivariable Adjusted 3^c^ | 0.83 [0.74, 0.93] | 0.0021 |
| a Includes adjustment for age, sex, race, ethnicity, education status, marital status, and work loss in last 7 days.  b Includes adjustment for all covariates listed in (a) plus household income, total number of adults in household and children in household.  c Includes adjustment for all covariates listed in (b) plus SNAP benefits, total amount spent on food in last week, use of anything other than income used to pay for necessities, and missing data indicators for the final two variables. | | |

Appendix Figure 1. Food Hardship (Binary) over Time by Race (Panel A), Income (Panel B), and Employment in last 7-Days (Panel C)
